# Bone-marrow edema estimation with a microwave imaging open-ended coaxial probe array in *ex vivo* bovine long bones: comparison with CT

**DOI:** 10.1186/s41747-026-00706-1

**Published:** 2026-04-13

**Authors:** Nadim Conti, Pablo Giaccaglia, Valentina Lidoni, Alessia Rosa, Luca Maria Sconfienza

**Affiliations:** 1 Politecnico di Milano, Piazza Leonardo Da Vinci 32, 20133 Milano, Italy; 2 Rilemo s.r.l., Via Don Sebastiano Colleoni 20, 23899 Robbiate, Italy; 3 Università Degli Studi di Milano-Bicocca, Piazza dell’Ateneo Nuovo 1, 20126 Milano, Italy; 4 Università Degli Studi di Milano, Via Festa del Perdono 7, 20122 Milano, Italy; 5 IRCCS Istituto Ortopedico Galeazzi, Via Cristina Belgiojoso 173, 20100 Milano, Italy

**Keywords:** Bone marrow, Cattle, Edema, Microwave imaging, Tomography (x-ray computed)

## Abstract

**Objective:**

We evaluated a microwave imaging prototype to estimate bone-marrow edema in *ex vivo* bovine samples, using computed tomography (CT) and injected volumes as references.

**Materials and methods:**

Seven samples comprising distal/proximal halves of two femurs and two humeri were imaged before/after injection of iodine-enhanced gel into drilled holes (Ø 2 mm, depth 8 mm; 25.13 μL/hole). A 4 × 4 open-ended coaxial probe (OECP) array was used over 16 positions with four 90° rotations, simulating a 16 × 16 array of 256 antennas. Raw S11 scattering parameters across a 2.25–3.00 GHz sweep were calibrated and inverted using the Stuchly model with target permittivity at 2.5 GHz. Each OECP antenna was characterized to define its effective penetration depth and equivalent lateral footprint. Upscaled permittivity maps were then generated *via* nonlinear interpolation, while fluid estimation was computed leveraging a refractive index mixing model.

**Results:**

Microwave estimated volumes were repeatable across the four rotations (mean coefficient of variation (CV) 2.8%; mean intraclass correlation coefficient (ICC) 0.999). Microwave estimates tracked the injected volumes with significant correlation (*r* = 0.81, *p* = 0.027) and moderate-to-good concordance (Lin’s concordance correlation coefficient [CCC] 0.70), with a small bias (+12.4%; 95% limits of agreement (LoA) -17.4% to +42.2%; mean absolute percent error 12.4%). Agreement with CT-derived volumes was weaker (*r* = 0.58, *p* = 0.169; CCC 0.23) with a larger bias (+40.9%; 95% LoA -10.3% to +92.2%).

**Conclusion:**

Microwave imaging allowed for detecting and quantifying small, μL scale, fluid inclusions within *ex vivo* long-bone halves, using OECP-based arrays.

**Relevance statement:**

Non-ionizing microwave imaging may represent a feasible complementary approach for imaging and quantitative assessment of fluid-related distributions in musculoskeletal tissues.

**Key Points:**

- A 16-element OECP microwave imaging array allows spatially resolved estimation of fluid-simulated inclusions in *ex vivo* bovine long bones.
- Experimental characterization of OECP antenna sensitivity enables resolution upscaling of dielectric permittivity maps.
- Fluid volumes derived from microwave permittivity maps show significant correlation with injected reference volumes (*r* = 0.81).

**Graphical Abstract:**

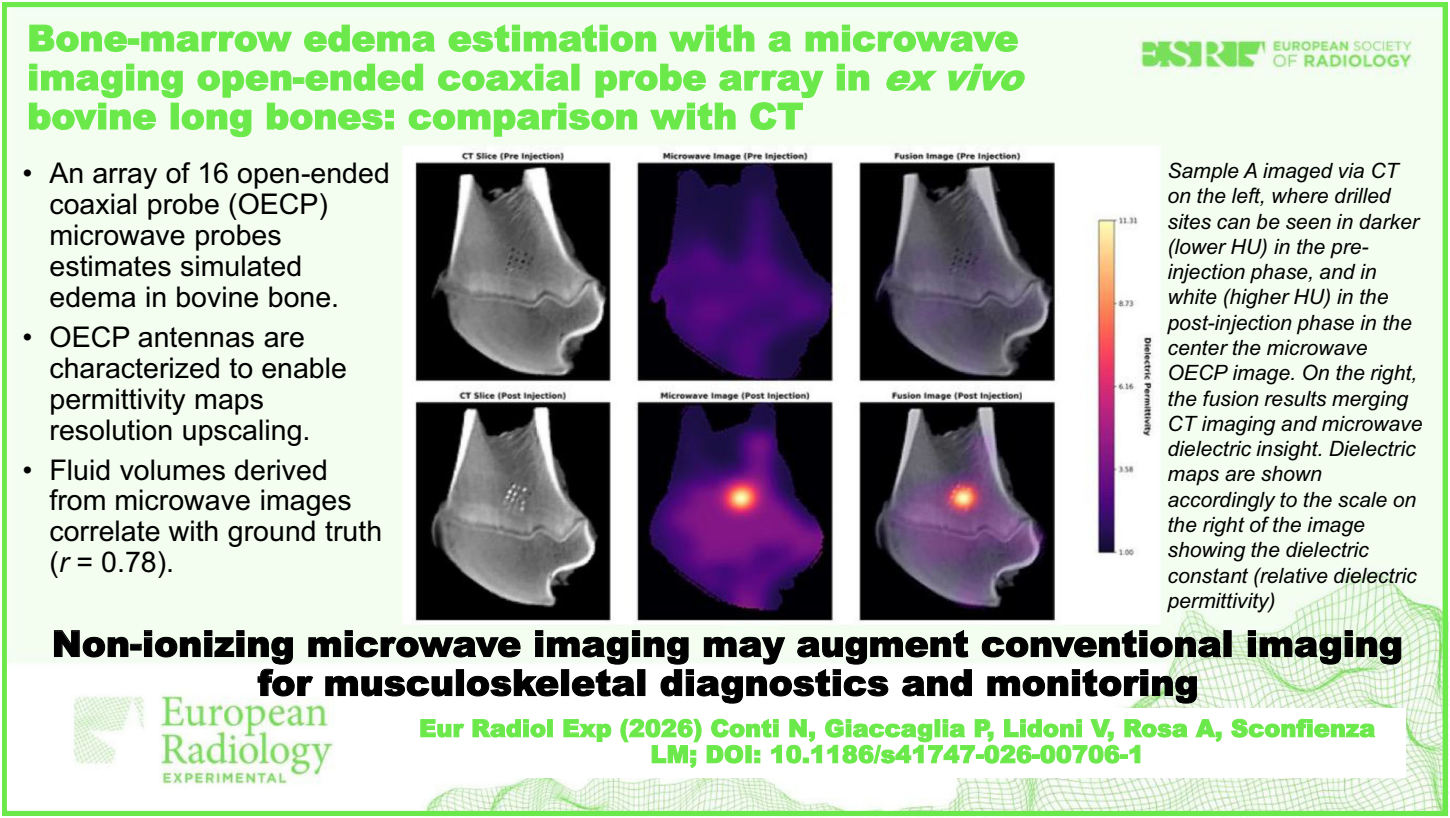

## Background

Musculoskeletal disorders rank among the leading causes of non-fatal disability worldwide [1]. While affecting individuals of any age, they frequently manifest with bone-marrow edema (BME) as a sensitive marker of disease activity. First described by Wilson et al in 1988 using magnetic resonance imaging (MRI) [2], BME (or bone marrow lesion) refers to abnormal fluid signals within the trabecular bone, characterized by ill-defined margins and high signal intensity, and appearing as intermediate T1 and high T2 signals within the marrow fat [3].

A broad spectrum of etiologies, including trauma, arthropathies (*e.g.*, rheumatoid arthritis), hypoperfusion, and neoplasia, can underlie BME, making it a non-specific but clinically valuable imaging sign [4]. MRI remains the gold standard, yielding a mean sensitivity/specificity of about 89.1% and 87.5% for various indications [5]. Recent studies, however, showed that even dual-energy computed tomography (CT) scanners can identify the lesion with great sensitivity and specificity both in acute and chronic settings [6, 7].

Despite the high utility of CT and MRI machines, they have limitations. Musculoskeletal imaging use has grown steadily, with utilization rates of radiography, ultrasound, CT, and MRI all increasing drastically over the last 20 years, leading to higher execution costs for the healthcare system [8]. Some studies indicate that the increase in nominal prices was the most significant contributor to healthcare spending growth, followed by increased utilization, inflation in imaging prices, shifts toward more advanced imaging modalities, and demographic changes [9]. Furthermore, MRI can trigger claustrophobia in sensitive individuals and may be limited in the presence of implants or devices such as certain pacemakers. Regarding CT, the chronic use of ionizing radiation is an increasingly important concern in relation to cancer risk in the general population.

Microwave imaging (MWI) exploits tissue dielectric permittivity differences and has shown promise for probing soft-tissue and bone properties without ionizing radiation [10]. However, much of the prior literature concerns microwave-based sensing applied to osteoporosis, osteogenesis monitoring, bone density changes after immobilization, and inflammation in arthritis or low-back pain, rather than true imaging [11–13]. By contrast, MWI systems capable of spatially resolving tissue features remain relatively unexplored in clinical musculoskeletal diagnostics, with few studies investigating the spatially resolved detection and quantitative assessment of localized fluid-related changes within mineralized bone structures.

Emerging MWI platforms aim to leverage spatially resolved dielectric contrasts to visualize internal fluid changes associated with bone marrow edema, inflammation, or traumatic injury, potentially complementing MRI and CT. However, most studies lack rigorous validation against CT or MRI ground truth, and experimental systems often remain at the phantom stage. Moreover, the majority of existing investigations focus on signal detection or qualitative contrast assessment, with limited emphasis on quantitative volumetric validation [10].

In the context of early-stage feasibility assessment, *ex vivo* models provide a controlled environment for evaluating the sensitivity of microwave imaging to well-defined dielectric perturbations under controlled conditions, thereby minimizing biological and physiological confounders. In this setting, computed tomography was chosen over radiographs to enable three-dimensional volumetric estimation of injected fluid within each drilled hole and preferred to MRI to avoid potential susceptibility artefacts arising from high contrast agent concentrations. CT offers high spatial resolution and reproducible geometric characterization of localized iodinated fluid inclusions within bone, making it a suitable quantitative reference standard for this proof-of-concept validation. Conversely, while MRI is the clinical reference modality for BME detection *in vivo*, its signal characteristics are sequence-dependent and less amenable to direct volumetric validation in simplified *ex vivo* models with known injected fluid volumes.

In this article, we present a novel microwave imaging prototype that employs a 16-element open-ended coaxial probe (OECP) array to estimate localized fluid-simulated dielectric inclusions in *ex vivo* bovine long-bone halves. By comparing quantitative permittivity-derived fluid volumes to CT-based measurements in drilled bone matrices, we aim to demonstrate the system’s sensitivity and accuracy as an initial step toward clinical translation.

## Materials and methods

No Institutional Review Board approval was required for this study. Seven samples, labeled from A to G, comprising distal and proximal halves of bovine femur (*n* = 4 halves) and humerus (*n* = 3 halves) were procured fresh from a local butcher (Camillo Valsecchi, Calolziocorte, Lecco) from northern Italy. The samples originated from a single 13-month-old male common dairy cow (Bos taurus). Bovine bone was selected as a biological surrogate for this study due to its macroscopic structural homogeneity and analogous dielectric contrast between the cortical shell and trabecular core to human tissue. Specimens were stored at -18 °C until 12 h before imaging, then thawed to 10 °C. All reported temperatures were verified with a calibrated Hickmicro Pocket 2 Infrared thermal camera. Flat surfaces were generated by cutting the samples longitudinally while cooled at -18 °C. No additional dehydration or chemical stabilization was performed.

A regular hole matrix (2 mm diameter, 8 mm depth, 25.13 μL per hole) was drilled on each flat face: samples A, B, and G (16 holes); E and F (20 holes); C and D (25 holes). Holes were spaced at 4-mm centers. A hair-gel/iodinated contrast mixture was prepared at 25% iodine contrast (iopamidolo, Iomeron 375, Bracco Imaging) 75% gel (water, denatured alcohol, vinylpyrrolidone/vinyl acetate copolymer, carbomer, benzyl alcohol, polyethylene glycol-40 hydrogenated castor oil, phenoxyethanol, sodium hydroxide, propyl glycol, disodium ethylenediaminetetraacetic acid, calcium chloride, magnesium gluconate, ferric chloride, copper sulfate, alpha-isomethyl ionone). Injections were performed via syringes while confirming total injected fluid by differential weighing on a calibrated Shimadzu UW420HV balance (*e* = 0.01, *d* = 0.001).

A 4 × 4 OECP array was driven by a calibrated and de-embedded vector network analyzer source at 0 dBm of nominal power (self-manufactured solid assembly; active flat surface), performing frequency sweeps from 2.25 to 3 GHz. Raw scattering (S11) measurements were recorded at each spatial position across a 16 × 16 grid obtained by moving the array in 4 cardinal positions. With an additional four probe rotations (0°, 90°, 180°, 270°) at each position, systematic errors were mitigated, resulting in a 16 × 16 effective measurement matrix. Each full acquisition required approximately 1 h and 10 min, comprising 30 s per 16-position scan and less than 10 min of total probe movement. The frequency-dependent complex permittivity was derived from raw S11 Scattering Parameters data using the Stuchly open-ended coaxial probe inversion model, following calibration with standardized reference measurements: open (air), short (aluminum foil), and two known dielectric load solutions (deionized water at 22 °C; 99.9% pure propylene glycol at 22 °C). Permittivity values at the target frequency of 2.5 GHz were extracted for analysis.

Two additional characterization procedures were performed on each OECP antenna. The center-to-edge sensitivity of each antenna was measured using known-permittivity cylinders (${\varepsilon }_{r}=4$, diameters 4–16 mm, 2 mm steps), verified in diameter and position by μm. Each sample was placed at the probe center and scanned systematically; the resulting response curve, reflecting increasing interaction with larger diameters, was used to derive a first factor of probe sensitivity. Side-to-side sensitivity was assessed by moving another set of uniformly large, flat rectangular reference samples laterally (± 15 mm, 1-mm steps) under the probes to derive a second factor of correction. The calibrated permittivity matrices obtained from the raw measurements were then upscaled to high-resolution permittivity maps through nonlinear interpolation using sigmoidal functions derived from the side-to-side and center-to-edge sensitivity curves. This interpolation approach ensured smooth and physically consistent transitions between matrix elements, accurately reflecting the spatial sensitivity profile of the OECP probe array.

To quantify the probe lateral footprint weight for fluid content estimation, the center-to-edge cumulative curve was inverted to recover the intrinsic radial sensitivity. Penetration depth was also characterized by measuring permittivity changes at varying probe-to-target distances and calibrated on homogeneous ABS and Carbon Fiber synthetic and water references, fitting the results in a visibility–thickness curve.

Specimens were mounted in a styrofoam jig to align flat faces parallel to the CT slice plane. Pre- and post-injection scans were acquired on a Revolution Ascend scanner, GE Healthcare, with 100 kVp, 80 mA, 3.0-mm slices, 0.94-mm pixels, and a “BONE” kernel.

For each microwave image reconstructed pixel, the post-injection permittivity was compared with the pre-injection baseline to estimate the aqueous inclusion fraction using a two-phase complex refractive index mixing model. Following the superposition of effects principle, as probe footprints strongly overlap, edema volumes were computed as the sum of all overlapping sensing regions. Depth weighting was applied via the effective penetration curve. Integration was restricted to an automatically derived region of interest encompassing contiguous post-injection permittivity increases. The final specimen-level fluid volume was obtained by summing voxel-wise (μL/mm^3^) contributions within the region of interest.

Coronal CT scans were considered to count contrast mixture-filled holes, blinded to the injected hole count. A hole was deemed positive if at least 50% the voxels in the hole had a value of 250 HU or greater, which is an approximation of the midpoint value between the two non-overlapping groups of voxel HU measurements of filled and unfilled holes in an equivalent threshold outcome span. For each positive hole, the volume was assumed fully filled and set to the nominal cylinder volume (Ø2 mm, depth 8 mm; 25.13 μL/hole).

Microwave-derived total fluid volumes were compared against CT-derived volumes and matched to the effective injected volumes (syringe differential weighting). For a better comparison with the actual probe’s viewing capacity, CT-derived volumes and injected weighted amounts were depth-rescaled to the probe’s effective penetration depth sensing curve, rather than the full 8-mm hole depth. Practically, each positive hole contributed its nominal cylinder volume scaled to the effective depth sensitivity curve of each probe. Injected volumes were equally scaled; therefore, all comparisons reflect the same interrogated equivalent slab. Microwave-derived volumes were then compared against these depth-rescaled CT and injection volumes using Pearson correlation, Lin’s concordance correlation coefficient (CCC), Deming regression, and Bland–Altman analysis. Systematic differences were tested with a paired Student *t*-test (or Wilcoxon signed-rank when non-normal). All confidence intervals were computed using bootstrap resampling.

Repeatability analysis was performed by exploiting the four 90° rotations acquired at each specimen position. Volume estimates were computed independently for each rotation, yielding four repeated measurements per specimen. Within-specimen repeatability was quantified using the coefficient of variation (CV) and intraclass correlation coefficient (ICC, two-way random effects, absolute agreement). Mean volume estimates across the four rotations were used for subsequent comparisons with reference volumes.

## Results

**Fig. 1:**
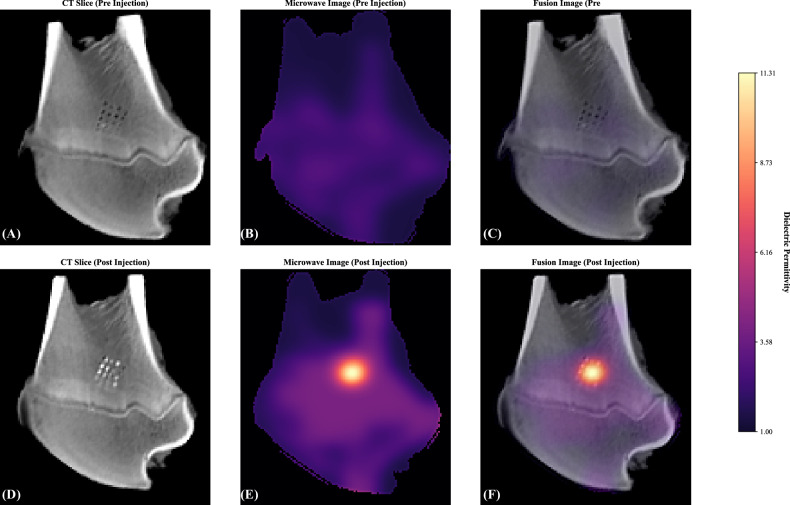
Multimodal imaging of specimen A before and after fluid injection. Top row (pre-injection): **A** CT slice showing drilled sites as darker regions (lower HU), **B** microwave permittivity map, **C** CT-MWI fusion image. Bottom row (post-injection): **D** CT slice showing contrast-filled holes as bright regions (higher HU), **E** microwave permittivity map showing increased signal at injection sites, **F** CT-MWI fusion image. The color bar indicates dielectric permittivity measured by the microwave device. CT, Computed tomography; MWI, Microwave imaging

All seven bone samples (A–G) were successfully imaged with the microwave imaging OECP device before and after fluid injection. The full pre-injection or post-injection protocol required about 1 h per sample. Figure 1 shows representative permittivity maps alongside corresponding coronal CT slices and fused images for sample A, highlighting clear increases in permittivity at the drilled-hole sites after injection when compared to pre-injection results.

**Fig. 2:**
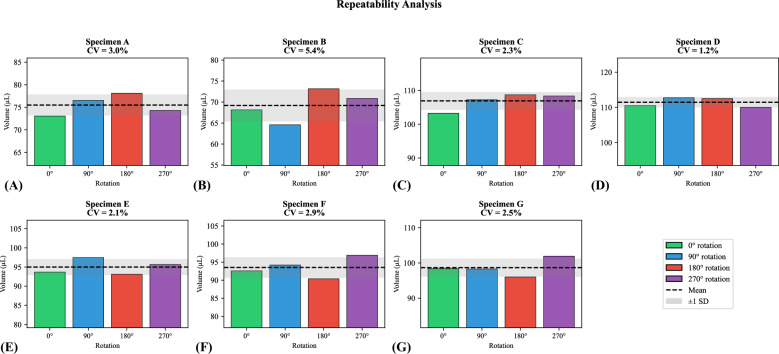
Repeatability analysis across all seven specimens (**A**–**G**). Bar charts display microwave imaging volume estimates (μL) obtained from four 90° rotations per specimen, demonstrating measurement stability under repositioning. Dashed lines indicate the mean volume; shaded bands represent ± 1 standard deviation (SD). All specimens showed consistent reproducibility with a coefficient of variation (CV) ranging from 1.2% to 5.4%, confirming that the volume derivation pipeline yields consistent results across repeated acquisitions

Repeatability analysis across the four 90° rotations demonstrated high measurement stability (Table 1 and Fig. 2). Within-specimen CV ranged from 1.24% to 5.36% (mean 2.77%), and ICC ranged from 0.997 to 0.9998 (mean 0.999), indicating strongly reproducible volume estimates under repositioning. Mean volume estimates across rotations were used for all subsequent comparisons.

**Table 1:** Repeatability analysis of MWI volume estimates across four 90° rotations per specimen

Specimen	Measured injected volume (µL)	Standard deviation (µL)	Coefficient of variation (%)	95% Confidence interval (µL)
A	75.50	2.26	2.99	[71.9, 79.1]
B	69.20	3.71	5.36	[63.3, 75.1]
C	106.90	2.50	2.34	[102.9, 110.9]
D	111.45	1.38	1.24	[109.3, 113.6]
E	94.99	1.98	2.08	[91.8, 98.1]
F	93.52	2.74	2.93	[89.2, 97.9]
G	98.65	2.44	2.48	[94.8, 102.5]

All specimens showed high reproducibility, with CV ranging from 1.24% to 5.36% (mean 2.77%) and ICC consistently above 0.99 (mean 0.999), confirming that the volume estimation pipeline is appropriate for quantitative assessment

*CV* Coefficient of variation, *ICC* Intraclass correlation coefficient, *MWI* Microwave imaging

Mean microwave estimates tracked the effective injected volumes with a statistically significant linear association (Pearson *r* = 0.811, *p* = 0.027; Fisher 95% CI 0.15–0.97; bootstrap 95% CI 0.38–0.99). Lin’s CCC indicated moderate-to-good agreement (CCC = 0.70; 95% CI 0.14–0.96), with high precision (*r* = 0.81) and good accuracy (bias correction factor = 0.86). Bland–Altman analysis indicated a small positive bias of +12.4% (95% LoA -17.4% to +42.2%; bootstrap 95% CI for bias +4.4% to +24.3%). Paired comparison confirmed a non-zero shift (Wilcoxon *p* = 0.016; mean difference +9.27 μL, bootstrap 95% CI + 3.70 to +17.11 μL). Accuracy summarized by MAPE was 12.4% (95% bootstrap CI 4.4–24.3%). Deming regression yielded a slope of 0.89 (95% CI 0.31–2.28), consistent with proportional agreement.

Regarding CT volume estimation, microwave imaging correlated moderately with CT (Pearson *r* = 0.58, *p* = 0.169; Fisher 95% CI -0.31 to 0.93; bootstrap 95% CI -0.46 to 0.98), with permutation testing confirming non-significance (*p* = 0.171). Lin’s CCC indicated poor agreement (CCC = 0.23; 95% CI -0.09 to 0.50), driven by a large location shift (Cb = 0.40). Bland–Altman analysis showed a positive mean bias of +40.9% (SD 26.1%; 95% LoA -10.3% to +92.2%; bootstrap 95% CI for bias +23.6% to +59.0%). In absolute units, the paired mean difference was +25.6 μL (*t*(6) = 4.99, *p* = 0.002), indicating consistent overestimation relative to CT. Notably, CT itself underestimated the injected reference by -18.6% (95% CI -27.9% to -9.3%), which partly explains the MWI-*versus*-CT discrepancy.

**Fig. 3:**
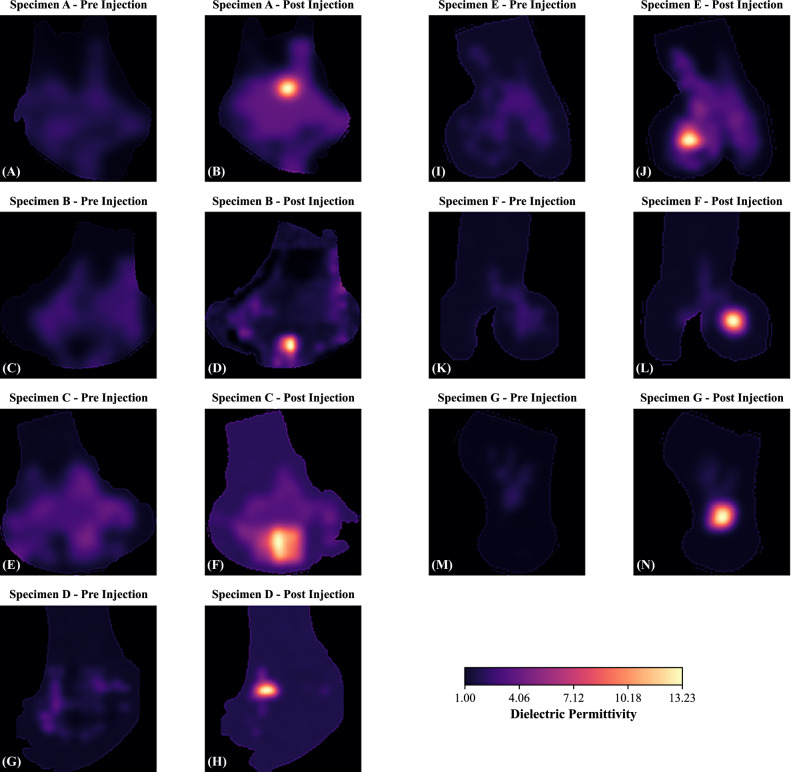
Pre-injection and post-injection pairs of the microwave imaging relative dielectric permittivity maps for the seven specimens (**A**–**G**), showing consistent detection of injected fluid across total injection volumes of 402–628 μL. Specimens **A**–**D** are shown on the left, and specimens **E**–**G** on the right. In all post-injection maps, localized increases in permittivity are observed at the drilled-hole injection sites, demonstrating sensitivity to μL-scale dielectric changes in *ex vivo* bone

Figure 3 shows the pre- and post-injection pairs of the microwave imaging relative dielectric permittivity maps.

Detailed numerical results regarding the matching between microwave volume estimation, syringe weighted volume and CT volume analysis are reported in Table 2.

**Table 2:** Detailed numerical results per sample

Specimen	Measured injected volume (µL)	Measured injected uncertainty (µL)	Scaled injected volume (µL)	CT estimated volume (µL)	CT scaled volume (µL)	MWI measured volume (µL)
A	402.12	115.94	67.86	326.73	55.13	75.50
B	402.12	115.94	67.86	402.12	67.86	69.20
C	628.32	181.16	106.03	452.39	76.34	106.90
D	628.32	181.16	106.03	552.92	93.31	111.45
E	502.66	144.92	84.82	376.99	63.62	94.99
F	502.66	144.92	84.82	301.59	50.89	93.52
G	402.12	115.94	67.86	376.99	63.62	98.65

From left to right: the sample ID, the measured injected volume with calibrated syringes, double-checked with a weight balance. The uncertainty of such volume. The scale measured volume to the equivalent measurable profile by the OECP probes. The CT estimated volume and relative scaled volume, and the Microwave Imaging measured volume of the edema liquid

*CT* Computed tomography, *MWI* Microwave imaging, *OECP* Open-ended coaxial probe

## Discussion

Mean MWI edema volume estimates tracked the injected (depth-scaled) fluid with statistically significant association (*r* = 0.81, *p* = 0.027) and moderate-to-good concordance (Lin’s CCC = 0.70), accompanied by a small, consistent positive bias of approximately +12%. Agreement with CT (depth-scaled) was weaker (*r* = 0.58, *p* = 0.169; CCC = 0.23) with a larger positive bias (+ 41%). This divergence is consistent with CT underestimation (-19% relative to injected volume) driven by partial-volume effects at the 2-mm hole diameter with 3.0 mm slice thickness, and the chosen conservative positivity protocol (≥ 250 HU in ≥ 50% of the cylinder with full-fill assignment for positives). CT provided a stringent but imperfect comparator at the μL scale, whereas the injected volume represented the delivered load and aligned more closely with MWI.

The four 90° rotations enabled a within-session repeatability assessment (Fig. 2 and Table 1). High reproducibility was observed (mean CV = 2.8%; mean ICC = 0.999), indicating that the volume estimation pipeline is stable under repositioning. While this is not a full test-retest study with independent acquisitions, the multi-rotation protocol provides evidence of measurement consistency that supports the reliability of the quantitative estimates.

**Fig. 4:**
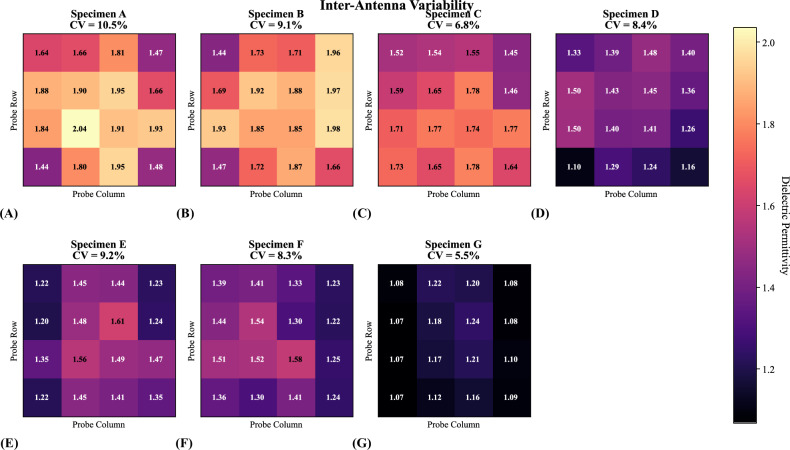
Inter-antenna variability analysis across all seven specimens (**A**–**G**). Each heatmap displays the mean baseline permittivity measured by each probe in the 4 × 4 array, averaged across all pre-injection acquisitions for that specimen. Individual specimen CV values quantify measurement consistency across the 16 probes. Overall mean CV = 8.2% (range 5.5–10.5%), confirming acceptable inter-antenna uniformity for quantitative volume estimation and supporting the reliability of the array for spatially resolved dielectric imaging. CV, Coefficient of variation

Inter-antenna variability was characterized across the 16-probe array (Fig. 4 and Table 3). The CV of inter-antenna measurements was 8.2% (range 5.5–10.5%), confirming acceptable uniformity across the array for quantitative volume estimation.

**Table 3:** Inter-antenna variability analysis showing baseline dielectric permittivity measured across the 16-probe array for each specimen

Specimen	Mean dielectric permittivity	Standard deviation	Coefficient of variation (%)
A	1.771	0.185	10.47
B	1.789	0.162	9.06
C	1.646	0.112	6.79
D	1.357	0.114	8.40
E	1.386	0.127	9.16
F	1.378	0.114	8.26
G	1.134	0.062	5.48

Values are averaged from all pre-injection acquisitions. Mean coefficient of variation of 8.2% (range 5.5–10.5%) indicates acceptable uniformity across the array for quantitative volume estimation

The current architecture relies exclusively on S11 reflection measurements, a monostatic design chosen for compactness and simplicity that is well-suited to early-stage validation of depth-scaled volume inference. The data elaboration pipeline was designed to respect the physics of the probe: raw S11 was inverted to complex permittivity, and spatial weighting was characterized experimentally (center-to-edge and side-to-side), from which an effective penetration depth and equivalent lateral radius were derived. This process allowed us to derive a cylindrical weighting kernel used both in interpolation and in quantitative fluid integration. As neighboring footprints overlapped extensively, volumes were computed over the union of sensing regions. A two-phase mixing law converted post- *versus* pre-injection permittivity differences to local fluid fraction. To achieve a realistic comparison with the MWI array viewing capacity, all references (CT and injected) were depth-harmonized to the probe’s effective penetration depth sensing curve.

The 13-month-old bovine samples used in this study predominantly consist of plexiform bone, which is denser and possesses a distinct microstructure compared to the Haversian systems typical of adult human bones [14]. Consequently, the dielectric permittivity and conductivity of these young, hydrated bovine samples are expected to be slightly higher than those reported for adult human bone in the standard literature [15] due to the higher water fraction in the plexiform matrix. Despite this absolute difference, the dielectric contrast between the cortical shell and trabecular core remains sufficiently analogous to human tissue to serve as a valid electromagnetic surrogate for validating electromagnetic imaging approaches.

This *ex vivo* model with drilled gel-filled holes represents a simplified surrogate of bone marrow edema, designed to provide controlled dielectric-contrast inclusions at known depths and volumes. It captures the essential physics of localized high-permittivity fluid within mineralized bone but does not replicate the diffuse margins, microvascular components, or tissue heterogeneity characteristic of pathological BME. The current acquisition protocol was designed for maximum precision in this proof-of-concept study rather than clinical speed. Translation to *in vivo* applications will require addressing additional factors, including marrow heterogeneity, temperature and hydration variability, cortical thickness differences, soft tissue attenuation, and patient motion.

The CT positivity of filled hole criteria (≥ 250 HU in ≥ 50% of voxels) was established through a quantitative analysis of the CT coronal images obtained before and after contrast injection. To determine the optimal discrimination threshold, each hole was segmented using a cylindrical volume of interest consistent with the drill diameter. The voxel intensity distributions were analyzed to calculate maximum, minimum and mean HU values for both empty (true negative) and filled (true positive) holes. The analysis identified two non-intersecting measurement groups. The maximum recorded intensity for any voxel within a true negative (empty) hole was 132 HU, whereas the minimum intensity recorded for a true positive (filled) hole was 388 HU. Additionally, the maximum recorded average intensity for any voxel within a true negative (empty) hole was 25 HU (standard deviation 73 HU), whereas the minimum intensity recorded for a true positive (filled) hole was 482 HU (standard deviation 132 HU). This resulted in a distinct separation gap of 256 HU between the two populations. The threshold of 250 HU was selected as it approximates the arithmetic mean of the max-min samples separation boundaries, 260 HU, and the arithmetic mean of the max-min average intensities separation of the two groups, 253 HU. Consequently, the selected cut-off is robust as any threshold value chosen within the interval [132, 388] HU would yield identical classification results, confirming that the distinction between empty and filled states is insensitive to minor fluctuations in voxel intensity around the chosen threshold.

Limitations include the small sample size (*n* = 7), which restricts generalizability despite each specimen containing multiple drilled holes (16–25 per sample) that collectively provided a broad range of injected volumes. The conservative CT positivity criteria (≥ 250 HU in ≥ 50% of voxels) ensured stringent reference validation but may have contributed to CT underestimation. Although CT segmentation was performed blinded to the injected hole count, independent validation by external observers was not performed in this proof-of-concept study. Additional limitations include manual assembly of the OECP array, inducing systematic deviations, and the requirement for a flat imaging interface. The small frequency bandwidth used for quantitation limits permittivity analysis, while the high frequency restricts signal penetration.

Future work will focus on addressing these limitations through staged and progressive validation efforts, including blinded, multi-center validation with independent CT segmentation by external observers and, ultimately, towards first *in vivo* feasibility studies enabled by S21-based configurations that relax the requirement for planar targets. Additional developments include multiplicative debiasing calibration learned on a training subset and validated on held-out specimens, faster hardware (parallel excitation/receive, motion control) and conformal arrays to improve contact and coverage, as well as larger frequency bandwidth measurements to refine depth weighting and dispersion modeling. Finally, a validation of MWI against MRI in animal tissue models can be investigated together with the change of antenna and probe geometry to enable S21 signal parameter analysis on top of the here discussed S11-only approach.

## Data Availability

Raw data supporting the findings of this study are publicly available at: https://github.com/rilemo-engineering/bone-marrow-edema-mwi-data.

## Abbreviations

BME: Bone-marrow edema
CCC: Concordance correlation coefficient
CT: Computed tomography
CV: Coefficient of variation
ICC: Intraclass correlation coefficient
LoA: Limits of agreement
MRI: Magnetic resonance imaging
MWI: Microwave imaging
OECP: Open-ended coaxial probe
